# Synergy of combined Doxycycline/TUDCA treatment in lowering Transthyretin deposition and associated biomarkers: studies in FAP mouse models

**DOI:** 10.1186/1479-5876-8-74

**Published:** 2010-07-30

**Authors:** Isabel Cardoso, Diana Martins, Tania Ribeiro, Giampaolo Merlini, Maria João Saraiva

**Affiliations:** 1 Molecular Neurobiology Unit, IBMC- Instituto de Biologia Molecular e Celular, Rua do Campo Alegre 823, 4150-180 Porto, Portugal; 2 Escola Superior de Tecnologia da Saúde do Porto, Instituto Politécnico do Porto, Rua Valente Perfeito 322, 4400-330 Vila Nova de Gaia, Portugal; 3 Amyloidosis Research and Treatment Center , Fondazione IRCCS Policlinico San Matteo and Department of Biochemistry, University of Pavia P. le Golgi, 19, 27100 Pavia, Italy; 4 ICBAS- Instituto de Ciências Biomédicas Abel Salazar, Largo Prof. Abel Salazar, 2, 4099-003 Porto Portugal

## Abstract

Familial Amyloidotic Polyneuropathy (FAP) is a disorder characterized by the extracellular deposition of fibrillar Transthyretin (TTR) amyloid, with a special involvement of the peripheral nerve. We had previously shown that doxycycline administered for 3 months at 40 mg/Kg/ml in the drinking water, was capable of removing TTR amyloid deposits present in stomachs of old TTR-V30M transgenic mice; the removal was accompanied by a decrease in extracellular matrix remodeling proteins that accompany fibrillar deposition, but not of non-fibrillar TTR deposition and/or markers associated with pre-fibrillar deposits. On the other hand, Tauroursodeoxycholic acid (TUDCA), a biliary acid, administrated to the same mouse model was shown to be effective at lowering deposited non-fibrillar TTR, as well as the levels of markers associated with pre-fibrillar TTR, but only at young ages.

In the present work we evaluated different doxycycline administration schemes, including different periods of treatment, different dosages and different FAP TTR V30M animal models. Evaluation included CR staining, immunohistochemistry for TTR, metalloproteinase 9 (MMP-9) and serum amyloid P component (SAP). We determined that a minimum period of 15 days of treatment with a 8 mg/Kg/day dosage resulted in fibril removal. The possibility of intermittent treatments was also assessed and a maximum period of 15 days of suspension was determined to maintain tissues amyloid-free. Combined cycled doxycycline and TUDCA administration to mice with amyloid deposition, using two different concentrations of both drugs, was more effective than either individual doxycycline or TUDCA, in significantly lowering TTR deposition and associated tissue markers. The observed synergistic effect of doxycycline/TUDCA in the range of human tolerable quantities, in the transgenic TTR mice models prompts their application in FAP, particularly in the early stages of disease.

## Introduction

Familial Amyloid Polyneuropathy (FAP) is characterized by the deposition of Transthyretin (TTR) amyloid fibrils in several organs, with special involvement of the peripheral nerve. Therapy is presently based on liver transplantation in selected patients, although progression of amyloid cardiomyopathy after liver transplantation is a serious and unsolved concern. FAP is characterized by early impairment of temperature and pain sensation in the feet, autonomic dysfunction leading to malabsorption and emaciation. Amyloid deposits can occur in any part of the peripheral nervous system, including nerve trunks, plexus and sensory and autonomic ganglia. TTR V30M is the most common TTR mutation associated with FAP but over 100 mutations are identified and associated with disease [1].

Animal models for a variety of disorders have been widely used to investigate mechanisms leading to disease and to assess possible therapeutic strategies. Therefore, a number of models have been created for FAP, including mice carrying the most prevalent TTR mutation, V30M, under the control of different promoters [2,3], and a highly amyloidogenic TTR variant, L55P [4]. The characterization of these models revealed early presence of non-fibrillar TTR that with aging evolved to TTR amyloid deposits [4], thus mimicking the human pathological characteristics, except for the lack of deposits in the peripheral nerve. Very recently, another FAP mouse model was generated, the V30M transgenic mice in a heat shock factor 1 (HSF-1) null background, representing a step forward in the study of this disorder; these mice represent an improved FAP model since animals showed TTR deposition in extra-neural and neural tissues, and recapitulated pathological findings in FAP. Amyloid deposition occurred in the peripheral and autonomic nervous systems and induction of pro-inflammatory cytokines, up-regulation of the receptor for advanced glycation end products (RAGE) and NF-κB activation were evident in the dorsal root ganglia and peripheral nerve presenting TTR deposits. Deposition did not occur in the brain and spinal cord; furthermore, a significant decrease in unmyelinated fibers occurred when fibrillar material was deposited in nerve [5].

While several reports describe the use of different therapeutic strategies in mouse models for neurodegenerative disorders such as Huntington disease (HD) [6], Parkinson's disease, Alzheimer's disease (AD) [7], Prion disease [8] and other, *in vivo *assessment of potential therapies has not been fully exploited in FAP mouse models. Doxycycline shown as a TTR fibril disrupter *in vitro *[9], when tested in transgenic TTR V30M mice, was capable of disaggregating amyloid deposits with concomitant decrease of metalloproteinase 9 (MMP-9), tissue inhibitor of metalloproteinase (TIMP-1), serum amyloid P component (SAP) and neutrophil gelatinase-associated lipocalin NGAL [10,11]. However, doxycycline was unable to remove non-fibrillar TTR or to lower non-fibrillar TTR-associated markers. On the other hand, Tauroursodeoxycholic acid (TUDCA), a biliary acid acting as a potent anti-apoptotic and anti-oxidant was also evaluated in TTR-V30M transgenic mice. Decreased apoptotic and oxidative biomarkers usually associated with TTR deposition, namely the ER stress markers BiP and eIF2alpha, the Fas death receptor and oxidation products such as 3-nitrotyrosine were significantly lowered. Most importantly, TUDCA treatment significantly reduced TTR toxic aggregates in as much as 75% [12]. However, TUDCA efficiency was only evident in young mice displaying non-fibrillar TTR deposits, i.e., in the absence of amyloid deposits. In the present work we assessed the efficiency of different doxycycline and TUDCA concentrations, either in individual or combined regimens, to lower TTR deposits and to affect molecular biomarkers associated with deposition.

## Material and methods

### Animals

All animals were kept and used strictly in accordance with national rules and European Communities Council Directive (86/609/EEC), and all studies performed were approved by the Portuguese General Veterinarian Board (authorization number 024976 from DGV- Portugal). Transgenic mice for human V30M TTR in a TTR null background, thereafter referred to as the TTR model, were kindly provided by Professor Suichiro Maeda from Yamanashi University. These animals were previously analyzed in our laboratory and ~60% of the animals over 1 year of age were found to have TTR deposition as amyloid, i.e., Congo red (CR)-positive material [4], having non-fibrillar TTR deposits at younger ages. Mice expressing human TTR V30M in a TTR null background heterozygous for the heat shock transcription factor 1 (Hsf1), thereafter referred to as the TTR/HSF mice model, were recently characterized and shown to have early non-fibrillar TTR deposition as compared to TTR mice, evolving to amyloid deposits in several organs and tissues including the peripheral nervous system [5].

For each model, 7- 15 animals with ages ranging from 16-25 months were treated with doxycycline (Sigma), at concentrations of 40 or 8 mg/Kg/day, administrated in the drinking water, protected from light and changed once a week. Mice were treated either for 1 month or 15 days and sacrificed just after the treatment period, or after an interruption of 15, 30 or 45 days (see table 1). Mice receiving water alone were used as controls.

**Table 1:** Different doxycycline treatment schemes, including duration of treatment, concentrations and mice models used

Treatment period	Dose (mg/Kg/day)	strain	Age (months)	Treatment MMP-9 positive	Control MMP-9 positive	Treatment CR positive	Control CR positive
1 month	40	TTR/HSF	19	2/10	6/8	0/10	5/10

15 days	40	TTR/HSF	26	2/10	7/8	2/10	3/7

15 days + 45 days interruption	40	TTR/HSF	20	8/10	6/8	5/10	4/8

15 days + 30 days interruption	40	TTR/HSF	23-25	0/10	10/10	0/10	8/10

15 days	8	TTR/HSF	16-18	1/7	9/10	1/7	8/10

15 days + 30 days interruption	8	TTR/HSF	16-18	8/12	4/4	7/12	3/7

15 days + 15 days interruption	8	TTR/HSF	20	4/10	7/8	1/10	4/8

15 days + 15 days interruption	8	TTR	22	2/15	6/8	2/15	4/8

Ratios between the number of animals which showed positive staining for MMP-9 and CR and the total number of mice used in each group are shown.

In other instances, TTR mice 17-21 months (7-9 animals) were treated in alternated cycles with doxycycline/TUDCA: doxycycline was administrated for 15 days at 40 or 8 mg/Kg/day followed by TUDCA (Calbiochem) administration, also in the drinking water, for 30 days at 500 or 50 mg/Kg/day; this regimen was performed twice in a total treatment period of three months. Non-treated mice or treated with the individual drugs for the same time period were used as controls.

Animals were killed after anesthesia with ketamine/xylazine. Esophagus, stomach, small and large intestines were immediately excised and processed. Tissues were fixed in 4% neutral buffered formalin and embedded in paraffin or immediately frozen, for light microscopy or for total protein extraction, respectively.

### CR staining

The presence of amyloid in tissue sections was investigated after staining with Congo red and observation under polarized light [13]. Briefly, deparaffinized tissues sections were incubated for 20 min with 80% ethanol saturated with NaCl followed by 0.5% Congo red in 80% ethanol saturated with NaCl, and analyzed under polarized light. Amyloid was identified by the characteristic green birefringence.

### Immunohistochemistry (IHC)

5 μm-thick sections were deparafinated in xylol and dehydrated in a descendent alcohol series. Endogenous peroxidase activity was inhibited with 3% hydrogen peroxide/100% methanol and sections were blocked in 4% bovine serum and 1% bovine serum albumin in phosphate buffered solution (PBS). Primary antibodies used were rabbit polyclonal anti- TTR (Dako, 1:1000), rabbit polyclonal anti-MMP-9 (Abcam, 1:1500), sheep polyclonal anti-SAP (a kind gift from the laboratory of Professor Pepys, Royal Free Hospital, London, 1:2000), rabbit polyclonal anti-biglycan (a kind gift from Dr P. Roughley, Shriners Hospitals, McGill University, Montreal, Canada,1:1000), goat anti-BiP (St Cruz, 1:50), rabbit polyclonal anti-Fas (St Cruz, 1:200), and rabbit polyclonal anti-3-nitrotyrosine (Chemicon, 1:500), which were diluted in blocking solution and incubated overnight at 4°C. Antigen visualization was performed with the biotin-extravidin-peroxidase kit (Sigma), using 3-amino-9-ethyl carbazole (Sigma) or diaminobenzidine as substrates. Semiquantitative immunohistochemistry analysis was carried out with the Universal Imaging system (NIH, Bethesda, MD) which performs automated particle analysis in a measured area, that is, the area occupied by pixels corresponding to the immunohistochemical substrate's color is counted and normalized relatively to the total area. Each slide used was analyzed in 5 different selected areas. Results shown represent % occupied area ± SD.

### Western blot

For western blot, frozen stomachs from 3 animals from each group were homogenized in PBS, pH 7.4 and total protein quantified using the Bio-Rad protein assay (Bio-Rad Laboratories); then 30 μg of each extract was run per lane, separated in a 12% SDS-PAGE gel and proteins transferred into a nitrocellulose membrane. Immunodetection was performed with rabbit polyclonal anti-BiP (Abcam, 1:500) and mouse anti-actin (1:1000, Sigma) antibodies; detection was performed with ECL^® ^(enhanced chemiluminescence; GE Healthcare). Quantification of BiP levels was performed by densitometry using the ImageQuant 5.1 software (Molecular Dynamics). Density values were normalized with β-actin expression. Results are presented as normalized density ± SD.

### Statistical analyses

All data examined were expressed as mean *± *S.E. Comparison between groups was made using the ANOVA test. A p value of less than 0.05 was considered statistically significant.

## Results

### 1. Doxycycline administration schemes

#### a. Different doxycycline periods in the TTR/HSF model

To address the importance of doxycycline as a TTR fibril disrupter and its use in future therapeutic approaches, we tested the initial described dosage (40 mg/Kg/day) in the TTR/HSF mice and decreased the period of treatment to 1 month.

As previously reported [11], CR staining is limited to very small and restricted areas, whereas MMP-9 staining although easily visualized, is usually either positive or negative; thus, in both cases we did not proceed to quantitative analysis, and instead we show the results as a percentage of positive relative to the total number of analyzed cases. Analysis of CR birefringence and MMP-9 positive cases indicated that doxycycline was able to act on amyloid deposits since no amyloid was found and MMP-9 positive slides were observed only in 20% of the mice (table 1). In the control group, MMP-9 and CR positive cases were found in 75% and 50% of the animals, respectively. We then repeated the experiment for a period of 15 days to determine if this drug was still able to act on amyloid in a shorter period of time. As reported in table 1, 15 days were sufficient to lower MMP-9 expression to negligible levels and to disaggregate amyloid, since only 20% of the animals were positive for these two markers, whereas non-treated mice showed considerable MMP-9 staining (87.5% of the mice) and amyloid (57% of the animals).

#### b. Testing various treatment intervals

Our studies were continued by performing doxycycline administration at 40 mg/Kg/day, followed by interruption of treatment for different periods of time, in TTR/HSF mice. A pause of 45 days after doxycycline administration for 15 days, did not maintain amyloid disaggregation as indicated by the observation of MMP-9 immunoreactivity and the presence of amyloid deposits in 80% and 50%, respectively, of the treated mice, comparable to the control group which showed values of 75% and 50% for these parameters, respectively. However, suspension of treatment for 30 days resulted in sustained negligible levels of MMP-9 and CR in all treated mice (table 1), while non-treated animals had significant amounts of both MMP-9 and amyloid in 100% and 80% of mice, respectively.

#### c. Testing different dosages of doxycycline

Due to the observation that 15 days were sufficient for doxycycline to act on amyloid deposits in mice tissues, we reasoned that a lower dosage would also be active. Doxycycline at 8 mg/Kg/day administrated to TTR/HSF mice for 15 days revealed sufficient to lower MMP-9 and to remove amyloid, as only 14.3% of the animals were positive for both markers (table 1). 90% and 80% were the values found in the control group for these markers, respectively (not shown). However, when using this dosage, suspension of treatment for 30 days resulted in MMP-9 immunoreactivity in 66.7% of the animals and in the presence of amyloid in 58.3% (table 1), similar to values found in the control group, respectively. In the light of these results, we next reduced the suspension period for 15 days and found positive staining for MMP-9 only in 40% of animals and congophilic material in only 10% of the mice, indicative of reasonably effective treatment at reducing the amyloid load.

**Figure 1:**
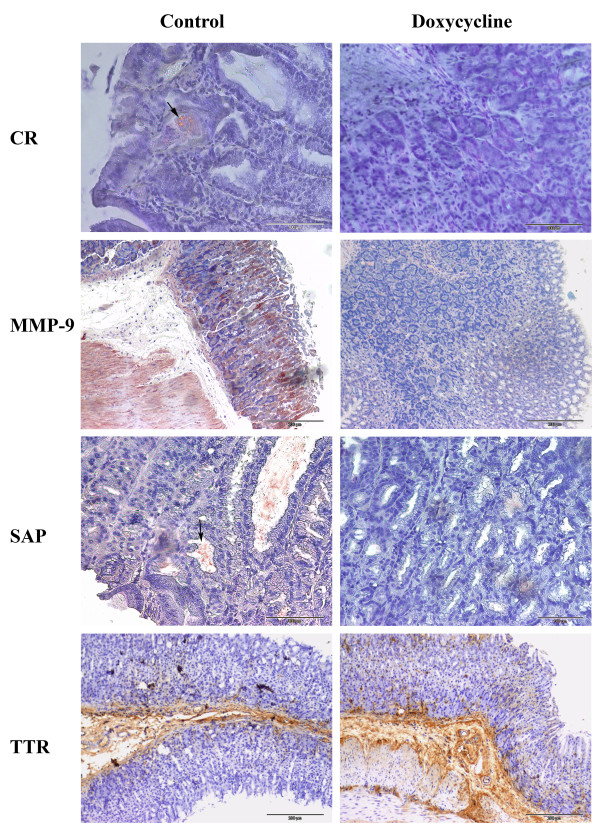
**Immunohistochemistry and Congo Red analysis of stomachs from TTR mice treated with doxycycline**. Doxycycline was administered in the drinking water (8 mg/Kg/day) for 15 days followed by a suspension period of 15 days (water alone) after which animals were sacrificed, (n = 15); control mice were given water alone (n = 11). The top 2 panels represent CR analyses (arrow pointing at CR birefringence in a non-treated mouse) and MMP-9 IHC. The bottom 2 panels are representative of SAP (arrow pointing at staining which co-localizes with amyloid in a non-treated mouse) and TTR IHC. Scale bar (CR and SAP = 100 μm; MMP-9 and TTR = 200 μm).

Evaluation of this regimen, i.e., 8 mg/Kg/day in 2 week cycles (15 days of treatment followed by 15 of interruption) was then performed in the TTR model. As reported in figure 1 and table 1, CR birefringence and MMP-9 staining revealed negative for the great majority of the treated animals, in opposition to the non-treated mice that showed positivity for both markers (see figure 1). Furthermore, SAP known to be associated to amyloid deposits was also negligible in treated mice, whereas non-treated animals showed, as expected, significant levels which mostly co-localized to congophilic amyloid deposits (figure 1). No significative changes were seen in non-fibrillar TTR deposits between treated and non-treated mice (figure 1).

### 2. Combined treatment with doxycycline and TUDCA

As we know that TUDCA is able to lower non-amyloid TTR deposits in young mice, we therefore hypothesized that a doxycycline/TUDCA combined treatment may be synergistic in lowering both fibrillar and non-fibrillar deposits.

#### a. High dosages

Doxycycline and TUDCA were cyclically administrated to TTR transgenic mice: doxycycline at 40 mg/kg/day for 15 days, followed by TUDCA at 500 mg/Kg/day for 30 days. This cycle was repeated twice; then tissues were analyzed for TTR deposition as well as for other FAP markers related to ECM remodeling, oxidative stress, cellular death, and ER stress, and compared with age-matched non-treated or single treated animals (either with doxycycline or TUDCA).

**Figure 2:**
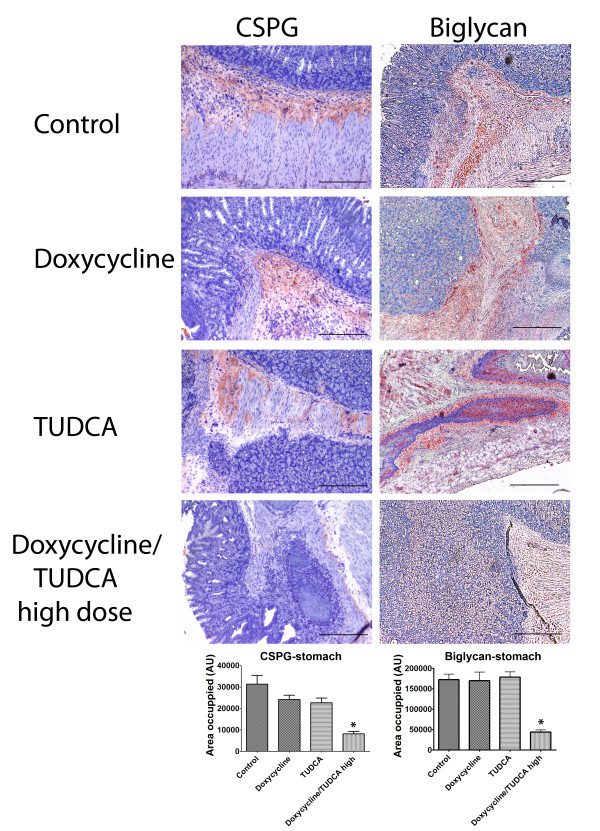
**Immunohistochemistry analyses for CSPG and biglycan in stomachs from TTR mice treated with doxycycline and TUDCA**. Groups of animals analyzed: control mice (**control**, upper row) (n = 7); then in the descending rows: mice receiving **doxycycline **alone (40 mg/Kg/day; n = 9); mice receiving **TUDCA **alone (500 mg/Kg/day; n = 7); mice receiving either high dosages of **doxycycline/TUDCA **(40 mg/Kg/day and 500 mg/Kg/day, respectively; n = 7). The combined scheme involved the administration of the referred dosage of doxycycline for 15 days, followed by the administration of the referred dosage of TUDCA for 30 days. The cycle was repeated twice; for the individual drug administration, during the interruption periods, mice were receiving water alone. Scale bar = 200 μm; **Histogram: **quantification of the levels of the referred markers, as described in material and methods.*P < 0.05.

Regarding evaluation of amyloid deposition and ECM integrity, we measured MMP-9 and CR positivity, CSPG and biglycan levels. Table 2 summarizes the results obtained for CR and MMP-9 staining in the stomach. No amyloid was observed in doxycycline treated animals, either in doxycycline/TUDCA or doxycycline regimens, which is expected and attributed to ability of this drug to disaggregate amyloid fibrils. The combined treatment had a pronounced effect on MMP-9 detection, as compared to doxycycline treatment alone (29% and 40%, respectively, table 2). TUDCA treatment did not alter the levels of this metalloproteinase which was equivalent to control non-treated animals. CSPG, previously established to be elevated in the TTR model in old mice presenting amyloid deposits [11], was now evaluated in the context of the combined doxycycline/TUDCA treatment. Results are depicted in figure 2 and showed that the combination of the 2 drugs was able to significantly decrease CSGP levels, whereas TUDCA or doxycycline alone were not able to change high CSPG levels, as compared to the control (figure 2, left panels). Biglycan is elevated both in FAP patients and in FAP mice models early in disease development, before amyloid is evident. Our previously reported data showed that doxycycline administration does not result in biglycan decrease probably because doxycycline being a fibril disrupter is not able to counteract pre-fibrillar TTR having no impact on biglycan levels. However, assessment of biglycan levels by IHC (figure 2, right panels) in the combined doxycycline/TUDCA treated mice revealed lower than in control (untreated mice), or in doxycycline alone (as expected) or in animals treated with TUDCA alone. Taken together, our observations indicated that combined administration of doxycycline/TUDCA is a strong therapy for ECM derangement, as all tested markers were restored to basal levels.

**Table 2:** Analysis of CR and MMP-9 patterns in the stomach of TTR mice

Mice group/Marker	CR positive	Positive MMP-9
Ctrl (at end)	50%	71%
(n = 7)		
Doxycycline/TUDCA	0%	29%
(n = 7)		
Doxycycline	0%	40%
(n = 9)		
TUDCA	50%	67%
(n = 7)		

The values refer to the percentage of positive related to total analyzed cases

**Figure 3:**
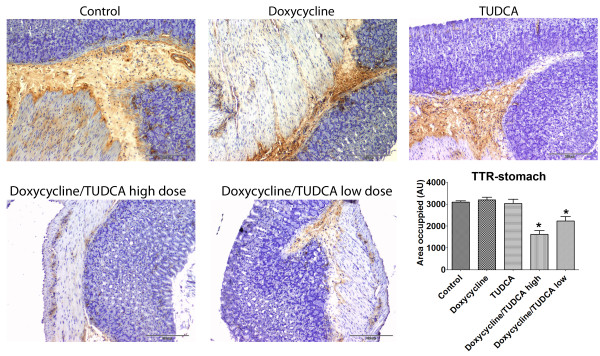
**Immunohistochemistry analyses for TTR in stomachs from TTR mice treated with doxycycline and TUDCA**. Groups of animals analyzed: in the upper row: **control **mice (n = 7); mice receiving **doxycycline **alone (40 mg/Kg/day; n = 9); mice receiving **TUDCA **alone (500 mg/Kg/day; n = 7); in the lower row: mice receiving either high dosages of **doxycycline/TUDCA **(40 mg/Kg/day and 500 mg/Kg/day, respectively; n = 7) or receiving low dosages of **doxycycline/TUDCA **(8 mg/Kg/day and 50 mg/Kg/day, respectively; n = 8). The combined scheme involved the administration of the referred dosage of doxycycline for 15 days, followed by the administration of the referred dosage of TUDCA for 30 days. The cycle was repeated twice; for the individual drug administration, during the interruption periods, mice were receiving water alone. Scale bar = 200 μm; **Histogram: **quantification of the levels of the referred marker, as described in material and methods.*P < 0.05.

**Figure 4:**
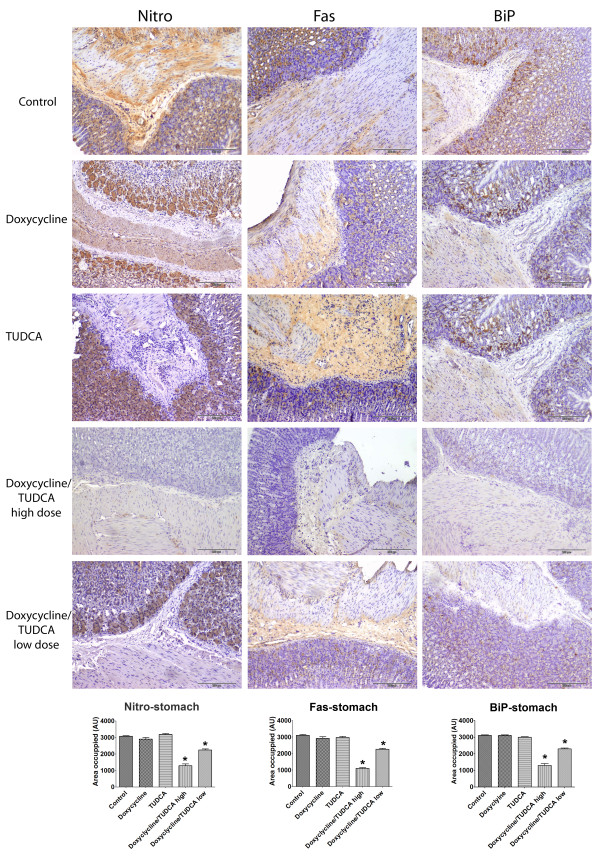
**Immunohistochemistry analyses for nitro, Fas and BiP in stomachs from TTR mice treated with doxycycline and TUDCA**. Groups of animals analyzed: control mice (**control**, upper row) (n = 7); then in the descending rows: mice receiving **doxycycline **alone (40 mg/Kg/day; n = 9); mice receiving **TUDCA **alone (500 mg/Kg/day; n = 7); mice receiving either high dosages of **doxycycline/TUDCA **(40 mg/Kg/day and 500 mg/Kg/day, respectively; n = 7) or receiving low dosages of **doxycycline/TUDCA **(8 mg/Kg/day and 50 mg/Kg/day, respectively; n = 8). The combined scheme involved the administration of the referred dosage of doxycycline for 15 days, followed by the administration of the referred dosage of TUDCA for 30 days. The cycle was repeated twice; for the individual drug administration, during the interruption periods, mice were receiving water alone. Scale bar = 200 μm; **Histogram: **quantification of the levels of the referred markers, as described in material and methods.*P < 0.05.

**Figure 5:**
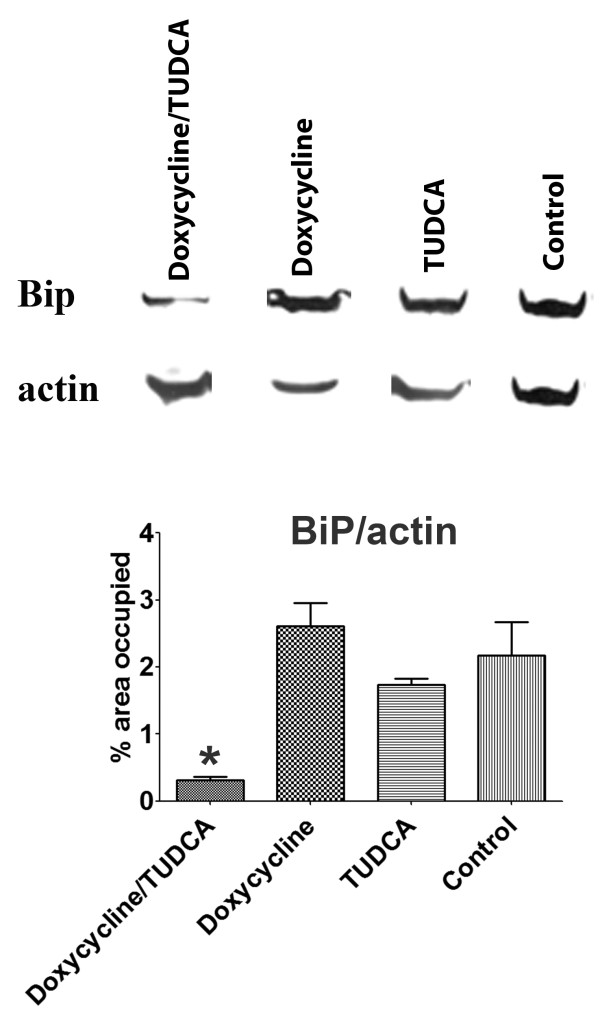
**Western blot analysis for BiP**. Stomachs from mice receiving combined cycled administration of doxycycline/TUDCA (40 mg/Kg/day and 500 mg/Kg/day, respectively; n = 3), mice receiving doxycycline (40 mg/Kg/day; n = 3), mice receiving TUDCA (500 mg/Kg/day; n = 3), and control mice (n = 3). Histogram: densitometry of BiP/actin band intensity, *P < 0.05.

At this point, it was imperative to assess levels of other markers, related to oxidative stress, ER stress and cellular death, markers not affected by doxycycline treatment or by TUDCA at old ages. Above all, it was necessary to assess extracellular deposition of TTR by immunohistochemistry in organs most affected with deposition. Figure 3 displays representative images of the results obtained and clearly shows that TTR deposition is significantly decreased in mice submitted to the high doxycycline/TUDCA dose combined treatment. On the contrary, mice subjected to either doxycycline or TUDCA administration presented, at the end of the experiment, TTR loads comparable to non-treated animals. As for nitrotyrosine, Fas and Bip, results indicated that all were significantly decreased only in mice receiving high doses of the combined treatment (figure 4). Analysis of other organs with characteristic TTR deposition, such as esophagus and intestine, revealed the same pattern, i.e., individual treatments with either doxycycline or TUDCA were not effective at lowering these markers, whereas the combined treatment was (not shown). Finally, and to confirm the IHC results, we also analyzed BiP levels by western blot, after protein extraction from frozen stomachs (figure 5), and confirmed the IHC results.

#### b. Low dosages

At this point we had verified the benefits of the cycled administration of doxycycline and TUDCA, and thus it was appropriate to test lower dosages of this combination. As for doxycycline and as described in the first sections of results, dosages of 8 mg/Kg/day were still effective in removing TTR amyloid. Regarding TUDCA, and although we did not perform dosage testing, we selected a dose of 50 mg/Kg/day as it corresponds to human admissible/tolerable dosages. Administration was performed as before, with combination of 2 cycles of doxycycline administrated for 15 days, followed by TUDCA for 30 days. Tissues with characteristic TTR deposition were processed and analyzed for the established markers. Because the dosage/period of administration/suspension for doxycycline had been well studied and determined, we were confident that this treatment resulted in the removal of amyloid and in ECM recovery, and thus we analyzed only MMP-9 reactivity. In the combined treatment, MMP-9 immunoreactivity was reduced in 33% of the mice, in opposition to 71% of positive control animals, indicative of tissue improvement at the ECM level in most animals.

In figures 3 and 4 we show representative images of the results with quantification of TTR deposition (figure 3) and of BiP, Fas and 2-nitrotyrosine levels (figure 4), as assessed by IHC. The quantification of staining clearly showed that levels of these 4 markers were significantly lowered in the combined treatment as compared to the non-treated or to individual doxycycline or TUDCA regiments, as easily concluded by comparing tissue staining intensity (histograms, figures 3 and 4). The decrease in TTR deposition and associated biomarkers was not as impressive in the lower dosages, which are equivalent to a clinical setting, as compared to the higher doses, indicating a dose response behavior that is still efficient.

## Discussion

The mechanism underlying FAP pathology is not completely understood and important insights arose from studies on FAP mice models and clinical samples. Transgenic mice, for instance, showed that TTR first presented as extracellular non-fibrillar deposits (lacking the characteristic CR birefringence) which were associated with increased levels of biomarkers also observed in biopsies of asymptomatic carriers of the V30M TTR mutation, such as 3-nitrotyrosine, Fas, BiP and others [12]. Old mice present both non-fibrillar deposits and TTR amyloid, indicative of a kinetic evolution between these two amyloidogenic species. Although this model does not present peripheral nerve involvement, the hallmark of the human pathology, it created the possibility to test the efficacy of several drugs *in vivo*. In fact, the knowledge acquired in the last years concerning the identification of intermediate species and the mechanisms underlying amyloidosis paved the way for the investigation of drugs capable of interfering with the amyloidogenic pathway, at different stages. One of such class of drugs are amyloid fibril disrupters such as doxycycline, expected to function in all amyloid fibril types, independently of their protein/peptide precursor.

In this work we proposed to use the TTR animal models to optimize doxycycline therapeutic regimen *in vivo*; in order to achieve this goal we tested three different drug concentration and different periods of administration, including different suspension times. We also used two different models, the TTR and the TTR/HSF model and concluded that a dose of 8 mg/Kg/day and a maximum of 15 days maintained fibril disaggregation.

In spite of the positive and exciting results obtained with this drug, doxycycline is not able to decrease all the FAP markers, and therefore we inferred that it would not perform optimally as a therapy for FAP. As TUDCA is effective in reducing TTR load in young TTR mice, in this work we also explored the potential of two drugs, doxycycline and TUDCA, and found a synergistic effect as combined cycled administration of both drugs was clearly beneficial to lower TTR deposition and all the biomarkers under study, whereas single individual drug administration failed to produce improvement of all of the markers evaluated. Recent data demonstrated clearly activation of the classical UPR pathways in FAP tissues not specialized in TTR synthesis but presenting extracellular TTR aggregate and fibril deposition [14]. More recently, we showed that TUDCA reduces BiP induction in TTR mice [12]. The ability of TUDCA to abolish ER stress was previously observed and reduction of BiP induction and caspase 12 activation were reported [15]; TUDCA is also able to protect against oxidative stress in tissues damaged by TTR deposition and thus, TUDCA can act at different levels, affecting TTR extracellular deposition. It has been suggested that a possible mechanism for TTR extracellular aggregation is the influence of secreted metabolites generated by oxidative stress and apoptosis on TTR aggregation [12]. By intervening at an early point in the apoptosis pathway and preventing the transfer of Bax to the mitochondria, TUDCA has the potential to save certain kinds of cells from early death (for a review see [16]). Doxycycline besides its ability to disrupt TTR amyloid fibrils, is known to be a matrix metalloproteinase inhibitor, and can in this way contribute to lower inflammation generated by the extracellular deposits [17]. MMP-9 is highly expressed at sites of inflammation and contributes to the pathogenesis of various chronic inflammatory diseases. In asthma, MMP-9 is upregulated and involved in remodeling processes [18-20]. Furthermore, Pycnogenol, an anti-inflammatory drug is able to attenuate signs of inflammation in asthma patients through reduction of MMP-9 secretion [21]. It is also established that the ER-mediated stress response contributes to inflammation [22]. In fact, an ER-stress-inflammation loop has been proposed [23]; in this way, TUDCA and doxycycline acting together may overcome each other limitations producing synergistic effects, by acting in a cascade that lowers inflammatory stress and ultimately results in TTR removal and tissue improvement.

Both doxycycline and TUDCA are safe drugs, and have been used in different clinical settings. TUDCA has been used in nutrition-associated liver disease [24], and other liver conditions, and highly assessed in the context of neurodegenerative disorders, especially in animal models of Huntington disease [16]. Doxycycline, besides its wide application as an antibiotic, is being used in human studies namely in aneurysm and vascular conditions [25-27] and also in multiple sclerosis [28].

Using the principle of interspecies drug dose scaling [29], and considering a standard body weight of 70 kg in man and 20 g in mouse, the ratio (W_human _/W_animal _)^0.7 ^= 302.58. Therefore the TUDCA dose of 50 mg/kg in mouse corresponds to 302.58 mg/day in man, and the TUDCA dose of 500 mg/kg corresponds to 3025.8 mg of TUDCA in man. The TUDCA recommended dose in man is 250-750 mg/day. Furthermore TUDCA is very well tolerated as the DL_50 _in mouse is 3.000 mg/Kg. Similarly, 8 mg/Kg/day of doxycycline in mouse corresponds to 48 mg/day in man, and the 40 mg/Kg/day dosage in mouse corresponds to 242 mg/day in man, largely within the human therapeutic ranges.

Based on this data we propose an open-label study for TTR amyloid patients with oral administration of doxycycline (200 mg/day) and TUDCA (750 mg/day), to evaluate efficacy, tolerability, safety and pharmacokinetics.

## Competing interests

The authors declare that they have no competing interests.

## Authors' contributions

IC participated in the design of the study, carried out the immunohistochemistry analysis for CSPG, biglycan and MMP-9, BiP western blot, performed the statistical analysis and drafted the manuscript. DM participated in animals sacrifice, organ collection and carried out immunohistochemistry analysis for TTR, Fas, nitro and BiP. TR participated in mouse colony maintenance, drug administration animal sacrifice and organ collection, and CR analysis. GM participated in the design of the study. MJS conceived the study, and participated in its design and coordination. All authors read and approved the final manuscript.
